# Regularly occurring bouts of retinal movements suggest an REM sleep–like state in jumping spiders

**DOI:** 10.1073/pnas.2204754119

**Published:** 2022-08-08

**Authors:** Daniela C. Rößler, Kris Kim, Massimo De Agrò, Alex Jordan, C Giovanni Galizia, Paul S. Shamble

**Affiliations:** ^a^Zukunftskolleg, University of Konstanz, Konstanz, 78464 Germany;; ^b^Department of Biology, University of Konstanz, Konstanz, 78464 Germany;; ^c^Department of Collective Behavior, Max Planck Institute of Animal Behavior, Konstanz, 78464 Germany;; ^d^John Harvard Distinguished Science Fellows Program, Harvard University, Cambridge, MA 02138;; ^e^Department of Biology, University of Florence, 50121 Florence, Italy

**Keywords:** dream, resting, rapid eye movement, salticid, sleep

## Abstract

Sleep and sleep-like states are present across the animal kingdom, with recent studies convincingly demonstrating sleep-like states in arthropods, nematodes, and even cnidarians. However, the existence of different sleep phases across taxa is as yet unclear. In particular, the study of rapid eye movement (REM) sleep is still largely centered on terrestrial vertebrates, particularly mammals and birds. The most salient indicator of REM sleep is the movement of eyes during this phase. Movable eyes, however, have evolved only in a limited number of lineages—an adaptation notably absent in insects and most terrestrial arthropods—restricting cross-species comparisons. Jumping spiders, however, possess movable retinal tubes to redirect gaze, and in newly emerged spiderlings, these movements can be directly observed through their temporarily translucent exoskeleton. Here, we report evidence for an REM sleep–like state in a terrestrial invertebrate: periodic bouts of retinal movements coupled with limb twitching and stereotyped leg curling behaviors during nocturnal resting in a jumping spider. Observed retinal movement bouts were consistent, including regular durations and intervals, with both increasing over the course of the night. That these characteristic REM sleep–like behaviors exist in a highly visual, long-diverged lineage further challenges our understanding of this sleep state. Comparisons across such long-diverged lineages likely hold important questions and answers about the visual brain as well as the origin, evolution, and function of REM sleep.

In many animals, sleep broadly consists of alternating periods of quiet and active sleep (i.e., periods of sleep without movements and periods of sleep with movements, respectively). During active sleep, brain waves appear similar to activity in the awake brain, a discovery first recognized in humans in 1953 based on observations of rapid eye movement (REM) during this sleep phase (1). REM sleep is further characterized by diffuse muscle atonia (sleep paralysis) that suppresses most body movements but displays smaller-scale muscle twitches in the limbs (2). Despite 70 y of research, the evolutionary origin and function of REM sleep remain elusive—due, at least in part, to a lack of taxonomic breadth in this field. This has begun to change over the last few decades, with evidence of REM-like sleep in nonavian reptiles and even cephalopods (3–5).

We recently described a nocturnal resting behavior in a jumping spider (*Evarcha arcuata*, Salticidae), where spiders suspend themselves upside down on a silk line to rest throughout the night (6). This characteristic and strictly nocturnal posture coupled with inactivity suggests that these salticids may be sleeping. While in this posture, adult spiders exhibited conspicuous phases with elevated activity that included stereotyped leg curling behavior as well as twitching of opisthosoma, spinnerets, and limbs. Crucially, while jumping spiders cannot move the lenses of their eyes, they can move their retinae to adjust their gaze—with muscles enabling axial, rotational, and directional movements of the entire retinal tube within the prosoma (7).

We hypothesized that these behaviors may be expressions of an REM sleep–like state. To address this, we carried out nighttime observations of young, newly emerged spiderlings, which lack pigmentation in their exoskeleton—enabling direct observation of retinal tubes (Fig. 1*B*).

**Fig. 1.**
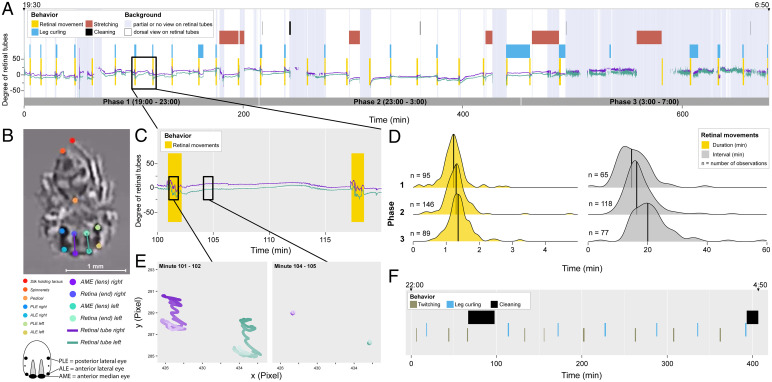
(*A*) Ethogram of a whole-night recording of a spiderling (E2108c, 09-03-2021 in the raw data). Colored boxes show observed behavioral sequences throughout the night. Green and purple lines show orientations of left and right retinal tube, respectively, extracted with automated video tracking (*SI Appendix*). Greyish blue shaded background areas indicate when the spider was not or only partially in dorsal view, limiting both manual and automated scoring of retinal movements (*SI Appendix*). (*B*) Screenshot from the video in *A* showing all tracked body points, with overlaid lines drawn from median lenses to the back of the retinal tubes. (*C*) Zoomed-in section of angular tracking overlaid on the manually scored retinal movements sequence highlighting the consistency between automated and manual scoring procedures. (*D*) Smoothed density plots showing durations (yellow) and intervals (gray) of retinal movement bouts. Black lines show medians. (*E*) Trajectory plots of left (green) and right (purple) ends of retinal tubes during 1 min of an REM sleep–like bout (*Left*) and 1 min of an in-between phase (*Right*; highlighted in *C*), with time progressing from dark to light shaded colors. (*F*) Example ethogram for an adult spider (E2102, 06-27-2021 in the raw data) showing regular periodicity of movement behaviors (twitching and leg curling).

## Methods and Results

We filmed *E. arcuata* spiderlings (*n* = 34, 1 to 9 d postemergence, approximate size = 1 to 2 mm) overnight using an infrared camera with an integrated light source (*SI Appendix*). Spiderlings were filmed between 7 PM and 7 AM, with nights divided into three 4-h phases to observe the effect of time (Fig. 1*A* and *SI Appendix*, *SI Extended Methods*). We manually scored behaviors, including time and duration of retinal movement bouts, leg curling, stretching, and cleaning behavior (definitions are in *SI Appendix*, *SI Extended Methods*). We also trained a neural network using DeepLabCut (*SI Appendix*), allowing us to estimate angular movement of each retina to illustrate retinal tube movements and visibility of the spider (Fig. 1 *A*–*C* and *E*, Movie S1, and *SI Appendix*, *SI Extended Methods*).

Periodic bouts of retinal movements in spiderlings were clearly visible (Movie S2) with consistent durations (median = 77.13 s, interquartile range (IQR) = 19.65 s, n_obs_ = 330, n_subj_ = 29) and regular intervals (median = 16.97 min, IQR = 6.56 min, n_obs_ = 260, n_subj_ = 17). Both the duration and the intervals increased significantly over the course of the night (duration: generalized linear mixed model (GLMM) analysis of deviance, χ^2^ = 13.82, *P* = 0.001, n_obs_ = 330, n_subj_ = 29, 11% increase; intervals: GLMM analysis of deviance, χ^2^ = 26.92, *P* < 0.001, n_obs_ = 260, n_subj_ = 17, 38% increase) (Fig. 1 *A* and *D* and *SI Appendix*, *SI Extended Methods*). Retinal movements were accompanied by body movements: characteristic leg curls (often accompanied by additional twitching) (Movie S3) or uncoordinated twitches of single limbs, the opisthosoma, and/or the spinnerets (Movie S4). Leg curls were associated with retinal movements in 100% of all observations (135/135). Conversely, leg curls were only present in 39.5% of all observed retinal movement bouts (135/342).

No retinal movements were observed during coordinated behaviors, such as stretching, readjustment of the supporting silk line (Movie S5), or cleaning sequences consisting of brushing movements (Movie S6). The highly coordinated nature of these movements suggests that animals were awake during these behaviors. Cleaning typically occurred shortly after REM sleep–like states, implying brief awakenings.

Adult *E. arcuata* (*n* = 3, one female, two males, approximate size = 7 to 8 mm) were filmed as above. In adults, retinal tubes were not visible due to pigmentation of the cuticle; however, we observed leg curling behaviors identical to those in spiderlings (Movie S7) with almost identical duration (adults: median = 83.63 s, IQR = 29.86 s, n_obs_ = 34, n_subj_ = 3; spiderlings: median = 83.87 s, IQR = 94.65 s, n_obs_ = 255, n_subj_ = 30). Additionally, like spiderlings, adults showed bouts of twitching (single limbs, spinnerets, opisthosoma, median = 90.67 s, IQR = 29.23 s, n_obs_ = 41) (Movie S8). Intervals between these REM-associated behaviors in adults were highly consistent (median = 27.96 min, IQR = 7.86 min, n_obs_ = 62, n_subj_ = 3) (Fig. 1*F*).

These salticids also overnight in nonhanging positions, either in a silk retreat or simply standing. We observed partial leg curling several times even in this standing position. Filming nonhanging adult spiders head on using the infrared camera resulted in direct observations of rapid retinal movements during partial leg curling behaviors (Movie S9)—evidence that leg curling behavior was consistently linked with retinal movements (i.e., an REM sleep–like state) in adults as well as in spiderlings.

## Discussion

This report provides direct evidence for an REM sleep–like state in a terrestrial invertebrate—an arthropod—with clear parallels to REM sleep in terrestrial vertebrates. The combination of periodic limb twitches and eye movements during this sleep-like state as well as the increase of duration of REM sleep–like bouts meets core behavioral criteria of REM sleep observed in vertebrates, including humans (2). While there is wide variation across mammalian species, it is noteworthy that intervals and durations of REM sleep–like bouts in salticids are quantitatively similar to those found in rats (average REM bouts in rats = 86 s) (8) and mice (intervals between REM bouts = 10 to 15 min) (9).

In jumping spiders, we observed twitches as well as larger-scale movements, including leg curls—broad leg contractions toward the sternum. The range in movements matches descriptions of incomplete, spatially mosaic muscle atonia (*SI Appendix*), known from vertebrate sleep suppression systems (2). Given the regularity of twitches and leg curls and their co-occurrence with retinal movements, both movement types appeared to be different expressions of the same active sleep–like phase.

Sleep and REM sleep in particular are mostly studied under laboratory conditions, limiting our understanding of sleep in nature. While difficult, studying sleep in natural settings is particularly important to understand its function. Sleeping animals are vulnerable due to increased arousal thresholds, limiting vigilance and defense (10). Some could even be conspicuous to predators, such as cephalopods that flash chromatophores during REM-like sleep (3). The considerable variation of sleep phases we observe in nature might, therefore, reflect species- and ecology-specific trade-offs, such as the unihemispheric non-REM sleep that allows marine mammals to sleep and swim simultaneously and great frigatebirds to sleep and fly simultaneously (11, 12). Jumping spiders of the species *E. arcuata* seem particularly suitable for such field-based sleep research, as they commonly rest at night by hanging exposed off the vegetation (6).

The complex visual and cognitive behaviors of salticids and their relatively small nervous system facilitate experimental tests of the role of visual experience in REM sleep–like retinal movements. Eye movement patterns during REM sleep have been hypothesized to be directly linked to the visual scene experienced while dreaming (13)—begging the deeper question of whether jumping spiders may be experiencing visual dreams. This raises a unique opportunity to test this “scanning hypothesis” in jumping spiders, where retinal movements can be observed. Since visual input can be controlled in jumping spiders early on (unlike in humans), retinal responses to repeated visual stimuli presented during the day might partially reappear during REM sleep–like states.

REM sleep may be expressed by eye or retinal movements only in visual animals but might be expressed differently in animals relying primarily on other sensory modes. For example, web-building spiders with poor vision but excellent vibration sensing may activate muscle groups associated with sensing vibrations during sleep as part of consolidating vibratory memory during awake states. Indeed, honeybees display different sleep phases, including an antennal movement phase and a deep sleep phase (with immobile antennae) linked to long-term memory formation (14). REM sleep can occur even in the absence of movable eyes (e.g., in owls and moles) (4). Similarly, nematodes and insects that twitch during resting (15) may show expressions of REM-like sleep. While sleep is ubiquitous in the animal kingdom, it remains to be demonstrated whether REM-like sleep is equally universal and how these sleep phases may be expressed in less visual species. Conversely, eye movement during REM sleep may be a unique feature of visual brains, with this convergent evolution suggesting some critical vision-specific functionality.

## Supplementary Material

Supplementary File

Supplementary File

Supplementary File

Supplementary File

Supplementary File

Supplementary File

Supplementary File

Supplementary File

Supplementary File

Supplementary File

## Data Availability

Scripts and code as well as the DLC project folder have been deposited in the Zenodo open data repository (https://doi.org/10.5281/zenodo.6616655) (16). All other study data are included in the article and/or supporting information.
