# Supplementary material for: Regularly occurring bouts of retinal movements suggest an REM sleep–like state in jumping spiders
Source: Proc Natl Acad Sci U S A. 2022 Aug 8;119(33):e2204754119. doi: 10.1073/pnas.2204754119 (PMC9388130; doi:10.1073/pnas.2204754119)
Supplement: Supplementary File [file pnas.2204754119.sapp.pdf]

PNAS

Supplementary Information for
Regularly occurring bouts of retinal movements suggest an REM sleep–like state in jumping spiders

Daniela C. Rößler, Kris Kim, Massimo De Agrò, Alex Jordan, C. Giovanni Galizia, Paul S. Shamble

Daniela C. Rößler
Email: daniela.c.roessler@gmail.com

This PDF file includes:

Supplementary text
Legends for Movies S1 to S9
Legends for Datasets S1 to S3
SI References

Other supplementary materials for this manuscript include the following:

Movies S1 to S9
Datasets S1 to S3

Supplementary Information Text

Extended methods. Additional information on animal collection, video recording, behavioral definitions, statistical analysis, automated tracking and data processing.

Animal collection and housing. We collected gravid *Evarcha arcuata* females as well as egg sacs from the field in Trier, Germany, between June and August 2021. Mothers usually stayed with the eggs/spiderlings until shortly before emergence (personal observation DCR). We separated the mothers from their egg sacs when they naturally left it. Egg sacs were kept in clear plastic containers (6×6×16 cm). The light regime was 12:12 (light source: Genaray SpectroLED Essential 365 Daylight, SP-E-365D) and the average temperature during captivity was 22°C.

Video recording. We used a low-budget night vision camera (ZIMOCE 3182-wifi) with a magnifier lens (Delamax magnifier lens, 67mm, +10) attached to film spiders at night between June and September 2021. Spiders were filmed opportunistically to minimize disturbance and to increase the chances of filming spiders in a hanging position with their retinal tubes visible. This means that spiderlings were not separated upon emergence but kept together. Of the usually around 20 spiderlings within one egg sac, several spiders suspended themselves from the lid of the box during the night. While this approach arguably hindered identification of individuals (with chances of filming the same spider more than once across different nights; more information about how spider identity was handled under “Statistical Analysis”), it increased the ability to film spiders from a dorsal view. The suspended nature of their resting meant that spiders could freely rotate on the silk over the course of the night. Thus, visibility of retinal tubes and other body parts could change throughout the recording influencing both the manual and the automatic tracking as follows: leg curls, for example, could still be observed clearly in a ventral position, which can result in ethograms recording a leg curl but no associated retinal movement (see Fig. 1A). To indicate this, we added the additional information in the background coloration of the graph when the spider was in full dorsal view (see data processing below for details). Equally, the manual scoring always included information on whether the retinal tubes were visible during any given behavior (see “Retinal View” in Dataset S1).

Manual scoring and behavioral definitions. The following behaviors were scored from videos using start and end frames.
Retinal movement (only scored in spiderlings): sudden movement of the retinal tubes, mostly, but not necessarily in synchronization. Retinal movements associated with active sleep include sudden changes in direction and last longer than one “side-to-side” sweep of the tubes. These retinal movements are either accompanied by stereotyped leg curls (see below) or sudden and uncoordinated twitching of spinnerets, limbs and/or the abdomen.
Leg curling (scored in adults and spiderlings): highly stereotyped leg position, patella stretched outwards with leg tips pointing towards sternum. Pedipalps also pointing towards sternum. Mostly involves all legs. Conspicuous spinneret and limb twitches occur frequently in this position. Retinal movement cooccur in all instances of leg curling. Leg curling behaviors were scored from initiation of the behavior until the legs returned to their original position.
Twitching (only scored in adults, as the size of spiderlings combined with a limited video resolution restricted the view on small structures such as the spinnerets): sudden bouts of movement including uncoordinated twitches of limbs, spinnerets and/or abdomen.
Cleaning (scored in adults and spiderlings): legs brush against each other or over the abdomen. Palps and legs may be cleaned with help of chelicerae. Coordinated movements imply an awake state.
Stretching (scored in adults and spiderlings): sudden extension of all legs on one side. Exclusively unilateral. When the side is stretched that includes the tarsus holding the silk, a swift switch of tarsus occurs. It is unclear whether stretching is associated with an active or quiet sleep-like state. While stretching itself (i.e., movement of the limbs) is most likely an awake behavior, the often long periods of motionlessness with slow but continuous decline in leg extension after the stretching behavior may be associated with a form of sleep. Stretching behaviors were scored from initiation of the behavior until regaining of the original position.

Since we did not test differences in arousal thresholds during presumed awake and sleep-like behaviors, it remains to be confirmed whether the observed REM sleep-like state meets the definition of “sleep”. The regularity of retinal movement bouts and durations as well as the observed twitches, however, can more likely be explained by a sleep-like state rather than by spontaneous bursts of arousal and subsequent wakefulness. Most compellingly, this is supported by the physiological nature of the leg curling behavior. Jumping spiders rely on hydraulic pressure for leg extension, which is produced by muscles in the prosoma. Observed leg curls most likely occur due to a drop in hydraulic pressure as a result of muscle atonia in the prosoma. A similar leg posture can only be observed in dead spiders. Consequently, leg curling constitutes an inactive locomotor system, which is unlikely to be associated with an awake state.

All frames were normalized to align the observations over the course of the night. As the duration of spiderlings being in the frame of the video varied largely across nocturnal filming, we decided to bin the observations into three equal phases of four hours (see main manuscript) after confirming that using normalized start time and phase support the same results in the analysis (see R code in the repository). Using phases instead of start time further had illustrative advantages.
All raw observation data can be found in Dataset S1.

Intervals were scored manually for adults and spiderlings as follows: For adults, intervals (from onset of REM bout to the next onset of REM bout) were scored using both leg curling and twitching behaviors as we associate both with REM-like sleep.
For spiderlings, we only extracted ‘retinal movement’ and scored intervals when the retinal tubes were clearly visible between bouts of phasic activity. We manually scored intervals as per the description above. Interval information can be found in Dataset S2.

Statistical analysis. Statistical analyses were carried out in R 3.6.2 (1). We used generalized linear mixed models (GLMMs) using the package *glmmTMB* (2). To test whether the phase of the night (or start time) had a significant effect on the dependent variables (duration & interval) we then applied an analysis of deviance to the resulting models using the package *car* (3). Subject ID was always included as a random effect. Spider identity was true within each video (night), and we treated each night as independent. While this means that some spiders may have been filmed more than once (across nights), the variance of SpiderID in the models was very low (Duration model: Variance of SpiderID = 0.002082, n_{subj} = 29; Interval model: Variance of SpiderID = 0.003793, n_{subj} = 17) and thus would not affect the data. Model fit was confirmed using the package *DHARMa* (4). All plots were generated using the package *ggplot2* (5). The complete R script for the data exploration and analysis is available from the Zenodo open science repository (https://doi.org/10.5281/zenodo.6616655).

Automated video tracking. *DeepLabCut™ Tracking*
DeepLabCutTM (DLC) (Version 2.1.8.2) (6,7), a 3D markerless pose estimation package, was used to train a ResNet-50 network to track various body points on *Evarcha arcuata* in overnight trial videos. We labeled, when visible, 11 points of interest (AME lens left, AME retina left, AME lens right, AME retina right, ALE left, ALE right, PLE left, PLE right, pedicel, spinnerets, tarsus holding the silk line) in 400 frames extracted (by k-mean clustering) from 10 01:00-16:00minute excerpts of trial videos (40 frames per excerpt).Video excerpts were selected to cover a variety of lighting conditions and spider behavior (leg curling, twitching, eye movement, cleaning, stretching). 95% of the labeled frames were used as the training set, 5% of the labeled frames were used as the test set. We trained a RestNet-50 network (8,9) with default parameters on the training set for 200,000 iterations with batch size 8. The evaluated network was found to have a train error of 2.46 pixels and a test of 3.44 pixels (in a frame size of 1920 pixels by 1080 pixels). This network was then used to track points of interest on trial videos with p-cutoff of 0.7. The DLC project is available from the Zenodo open science repository (https://doi.org/10.5281/zenodo.6616655).

Data processing. *Matlab*
Tracking data from DLC was then filtered using Matlab (Version 2020a). For each tracked point of interest DLC outputs position (x,y) and a confidence score (continuous from 0 to 1) for every timepoint (i.e., for every video frame). To reduce tracking-based error, we first removed

all low-confidence results for each point of interest (confidence < 0.90). The remaining high-quality points were then filtered as follows. First, for the x and y values for every point of interest, small gaps (gap size < 10 frames) were filled using the last high-confidence value. Position data was then smoothed twice with a moving window filter using the built-in Matlab "smooth" function (initial filter window size = 50; secondary filter window size = 20). This data was also used to determine the orientation of the hanging spider relative to the camera, as points of interest would be lost as the spider rotated away from the ideal dorsal perspective (see background in Fig. 1A).

We then transformed position data from the reference frame of the video to an animal-based reference frame—specifically, we converted overall position (x,y) to the approximate gaze angle of each eye within the head (θ_{left}, θ_{right}). For each time point, the right AME lens was set as the origin, and then all other points rotated so that the left AME lens was at 0º in polar coordinate space. The angles of the left and right AME retinae were then calculated relative to each lens, so that $\theta = 0$ represents a "straight-ahead" gaze and +/- angular values as right/left shifts. Note that these approximate gaze-angle calculations were used to allow us to determine when eye movements occurred, the general direction of such movements, and how movements compared between eyes. Critically, reconstructions of 3D positions were not possible from our 2D videos, thus these angles do not represent true gaze angles. The associated Matlab code is available from the Zenodo open science repository (https://doi.org/10.5281/zenodo.6616655).

Limitations of DLC data. Within the scope of this manuscript, data from DLC was used mainly for illustrative purposes and to confirm the observations that retinal tubes show regularly occurring bouts of movement (see Fig. 1A, 1C, 1E). Videos differed in lighting, in the number of spiders visible throughout the night and were limited in video resolution. Together with the rotation of spiders in the hanging position, these factors largely restricted a broad-scale use of automated video tracking for analytical purposes. Consequently, DLC was only used on one video with one spider continuously visible throughout the night (E2108c, 09-03-2021, Dataset S3) confirming manually scored retinal movement bouts and illustrating information on spider position. Generally, a 2D projection of retinal movements can be extracted using our trained neural network. In the future, we will use (and improve) this network on standardized, higher resolution videos, which will allow us to investigate in detail how retinas move during REM-like sleep.

Movie S1 (separate file). Automatically tracked landmarks, including retinal tubes, of a spiderling during a REM-like bout (Automated tracking using DeepLabCut).

Movie S2 (separate file). One hour of sleep in a spiderling at 64x speed demonstrating regular intervals and durations of REM-like bouts.

Movie S3 (separate file). Characteristic leg curling behavior coupled with retinal movements in a spiderling during REM-like bout.

Movie S4 (separate file). Characteristic twitching behavior coupled with retinal movements in spiderlings during REM-like bouts.

Movie S5 (separate file). Stretching behavior in a spiderling and an adult demonstrating unilateral leg extensions as well as tarsal switch in the adult.

Movie S6 (separate file). Cleaning behavior in a spiderling and an adult demonstrating coordinated brushing behavior.

Movie S7 (separate file). Characteristic leg curling behavior in adults.

Movie S8 (separate file). Characteristic twitching in adults affecting abdomen, single limbs and/or spinnerets.

Movie S9 (separate file). Partial leg curling behavior in a standing sleep position and frontal view on an adult during partial leg curling behavior demonstrating clear retinal movements.

Dataset S1 (separate file). Raw observation data

Dataset S2 (separate file). Manually scored interval data

Dataset S3 (separate file). Retinal movement data for spiderling E2108c, 2021-09-03, based on automated tracking

SI References

1. R. C. Team, Others, R: A language and environment for statistical computing (2019).
2. A. Magnusson, *et al.*, Package “glmmTMB.” *R Package Version 0. 2. 0* (2017).
3. J. Fox, S. Weisberg, *An R Companion to Applied Regression* (SAGE Publications, 2018).
4. F. Hartig, DHARMa: residual diagnostics for hierarchical (multi-level/mixed) regression models. R package (2017).
5. H. Wickham, *ggplot2: Elegant Graphics for Data Analysis* (Springer, Cham, 2016).
6. A. Mathis, *et al.*, DeepLabCut: markerless pose estimation of user-defined body parts with deep learning. *Nat. Neurosci.* **21**, 1281–1289 (2018).
7. T. Nath, *et al.*, Using DeepLabCut for 3D markerless pose estimation across species and behaviors. *Nat. Protoc.* **14**, 2152–2176 (2019).
8. E. Insafutdinov, L. Pishchulin, B. Andres, M. Andriluka, B. Schiele, DeeperCut: A Deeper, Stronger, and Faster Multi-person Pose Estimation Model in *Computer Vision – ECCV 2016*, (Springer International Publishing, 2016), pp. 34–50.
9. K. He, X. Zhang, S. Ren, J. Sun, Deep residual learning for image recognition in *2016 IEEE Conference on Computer Vision and Pattern Recognition (CVPR)*, (IEEE, 2016) https:/doi.org/10.1109/cvpr.2016.90 (March 17, 2022).
